# Effects of implementing a clinical pathway on antibiotic prophylaxis for patients who underwent an elective surgery

**DOI:** 10.1038/s41598-022-24145-1

**Published:** 2022-11-23

**Authors:** Somin Park, Sooyeon Kim, Hong Bin Kim, Sang Woong Youn, Soyeon Ahn, Kidong Kim

**Affiliations:** 1 Office of Quality Improvement and Process Innovation, Seoul National University Bundang Hospital, Seongman-si, Gyeonggi-do Republic of Korea; 2 Medical Research Collaborating Center, Seoul National University Bundang Hospital, Seongnam-si, Gyeonggi-do 13620 Republic of Korea; 3 Division of Infectious Diseases, Seoul National University Bundang Hospital, Seoul National University College of Medicine, Seongnam-si, Gyeonggi-do Republic of Korea; 4 Department of Dermatology, Seoul National University College of Medicine and Seoul National University Bundang Hospital, Seoul, South Korea; 5 Department of Obstetrics and Gynecology, Seoul National University Bundang Hospital, Seongnam-si, Gyeonggi-do 13620 Republic of Korea

**Keywords:** Health policy, Health services

## Abstract

A reduction in the unnecessary use of antibiotic prophylaxis can prevent antibiotic resistance and adverse drug events. We aimed to evaluate the effects of implementing clinical pathways (CPs) on adherence to a systematic and appropriate duration of antibiotic prophylaxis. We identified 61 eligible CPs and a total of 44,062 patients who underwent elective surgeries associated with CPs. The Poisson mixed model with an interrupted time-series analysis frame was applied to the patient-level data. This enabled a comparison of the adherence rate before and after CP implementation. Furthermore, we examined the effect of application or completion of CP on the adherence rate after implementation. Adherence to the antibiotic prophylaxis guideline substantially increased (incident rate ratio [IRR] 8.05; 95 confidence interval [CI] 2.64–24.55), compared with that before implementation. Following the implementation into the electronic entry system, we observed an improved adherence not only in CP completion but also in attempted CP execution (IRR of the executed but not completed cases 1.54; 95% CI 1.17–2.04; IRR of the executed and competed cases, 1.94; 95% CI 1.4–2.69). The implementation of CP into the electronic prescribing system was associated with a significant increase in the appropriate use of antibiotic prophylaxis among patients who underwent elective surgeries. The results suggest that a computer-assisted CP system for electronic health records could improve antibiotic adherence without significant expense.

## Introduction

The estimated global volumes of surgeries in 2004 and 2012 were 234.2 million and over 300 million, respectively^1^. According to a meta-analysis, surgical site infection (SSI) occurred in approximately 12% of all patients who underwent surgery^2^. Patients with an SSI have a higher risk of death than those without SSI^3,4^. Considering the ever-increasing volume of surgery, SSI prevention plays an important role in patient care. The estimated cost of SSI was $20,785 per patient care and $3 billion per year in the United States in 2012^5^. Therefore, optimal care of patients undergoing surgery and the prevention of SSI are imperative even in terms of their economic impact.

Antibiotic prophylaxis refers to the use of antibiotics before surgery to prevent surgery-related infections, including SSI. It reportedly reduces SSI in various surgeries^6–8^. According to the American Society of Health-System Pharmacists Therapeutic Guidelines, prophylactic antibiotics should be administered within 60 min, prior to a surgical incision and should be continued for less than 24 h postoperatively^9^. However, the antibiotic prophylaxis guideline is not followed frequently in clinical situations^10–12^. For example, a German multicenter study reported adherence to the guideline in 71% of the cases^10^. A study on pediatric surgical cases reported an adherence rate of 40%^11^. In contrast, an Australian study on patients with breast surgery reported an adherence rate of 13%^12^. Various reasons contribute to the aforementioned low adherence rates. Incorrect wound classification, uncoordinated process, and the exaggerated perception of SSI risk by a physician were the major causes for non-adherence^11,12^.

Proper adherence to antibiotic prophylaxis guidelines can lead to an improvement in the clinical outcomes. According to a study that used Taiwan claims data, adherence to the antibiotics prophylaxis guideline was associated with a shorter length of stay, fewer medical expenses, and lower 30-day readmission rates^13^. Similarly, an Australian retrospective study concluded that non-adherence increased the risk of SSI^14^. In addition, the prolonged use of prophylactic antibiotics correlated with an increased risk of acquiring antibiotic resistance^15^.

The clinical pathway (CP) is a tool to improve the quality of healthcare by standardizing the care process^16^. CP reportedly increased the adherence to an antibiotic prophylaxis guideline in a particular disease setting^17–19^. However, the systematic effect of CP implementation on adherence to an antibiotic prophylaxis guideline in a general clinical setting is rarely evaluated.

The objective of this study was to examine the long-term effects of CP integrated in an electronic health record (EHR) system on adherence to an antibiotic prophylaxis guideline. Our goal was to provide estimates of systemic changes over various diseases.

## Results

### Characteristics of the clinical pathway and patients

**Figure 1:**
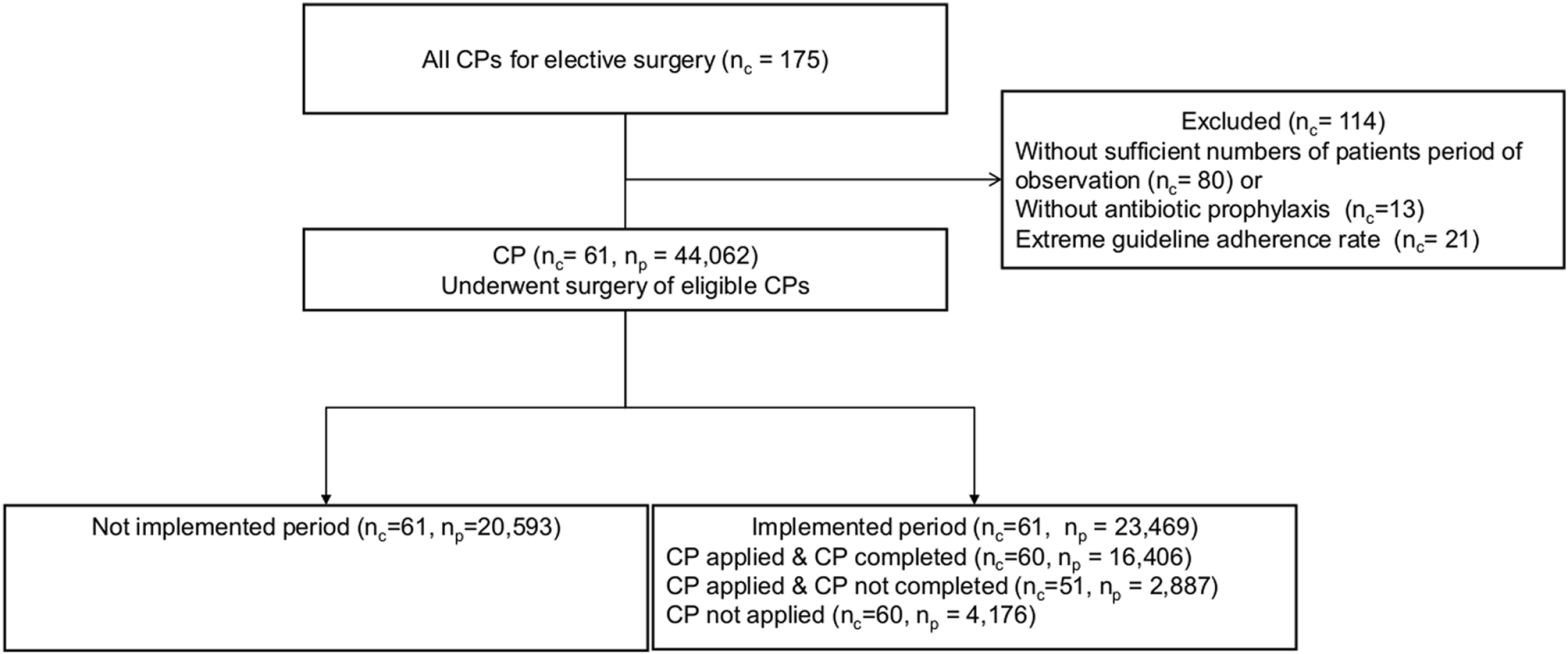
Flowchart showing the research methodology.

**Figure 2:**
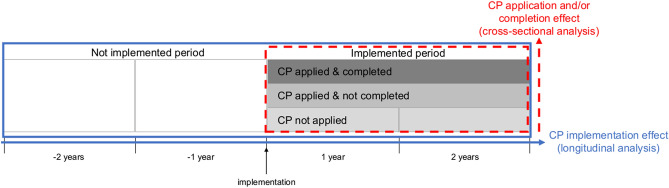
Observed adherence rate across the study period, with each line representing an individual clinical pathway.

A total of 44,062 patients were identified (Fig. 1). The median number of eligible patients per CP was 339 (Table 1). The median age of the patients was 45 years; 60% were women. The number of patients according to the relative admission date increased over time. CP was applied to 19,293 (82%) of the 23,469 patients, admitted after the implementation. Of these patients, 16,406 (85%) competed the CP process (Table 1). Figure 2 outlines the adherence rates across the study period.

**Table 1:** Characteristics of the clinical pathway and patients.

Clinical pathway (CP) (n_c_ = 61)	
**Implementation year, n (%)**	
2006	1 (2)
2007	2 (3)
2008	9 (15)
2009	7 (11)
2010	16 (26)
2011	7 (11)
2012	11 (18)
2013	4 (7)
2014	1 (2)
2015	3 (5)
**Recommended length of stay, days, n (%)**	
1	24 (39)
2	2 (3)
3	17 (28)
4	8 (13)
5	3 (5)
6	3 (5)
7	1 (2)
8	1 (2)
9	1 (2)
10	1 (2)
**Recommended duration of prophylactic antibiotics, day, n (%)**	
1	17 (28)
2	16 (26)
3	28 (46)
Number of eligible patients per CP, median (min–max)	339 (53–5277)
**Patients (n**_**p**_** = 44,062)**	
Age, median (min–max)	45 (0–99)
**Gender, n (%)**	
Male	17,643 (40)
Female	26,419 (60)
**Relative admission date to CP implementation date, n (%)**	
− 2 year ~ − 1 year	9690 (22)
− 1 year ~ CP implementation	10,903 (25)
CP implementation ~ + 1 year	11,451 (26)
+ 1 to + 2 year	12,018 (27)
Number of patients whom CP was applied to	19,293 (44%)
Number of patients whom CP was completed for	16,406 (37%)

### Effect of CP implementation on the adherence rate

According to the univariable and multivariable analysis, the adherence rate slightly increased a year before CP implementation. However, it sharply increased post-implementation (IRR at − 1 ~ 0 year, 2.26; 95% confidence interval [CI] 0.89–5.75; IRR at 0 ~ 1 year, 8.05; 95% CI 2.64–24.55). The enhanced effect of CP was maintained throughout the post-implementation period (IRR at 1 ~ 2 year, 7.76; 95% CI 2.47–24.4) (Table 2). The adjusted adherence rates were 7.6% (95% CI 6.33–9.13%), 17.2% (14.5–20.5%), 60.9% (51.3–72.2%), and 58.8% (50.0–70.0%), at − 2 ~ − 1 year, − 1 ~ 0 year, 0 ~ 1 year, and 1 ~ 2 year, respectively.

**Table 2:** Effects of clinical pathway (CP) implementation on the adherence rate (n_p_ = 44,062), and the incidence rate ratio (IRR) from the univariable and multivariable mixed-effect Poisson model with random effects.

Variable		Univariable		Multivariable	
		IRR (95% CI)	p-value^1^	IRR (95% CI)	p-value^1^
Relative admission date to CP implementation date	− 2 years to − 1 year	1	< 0.001	1	< 0.001
	− 1 year ~ 0	2.27 (2.08–2.46)		2.26 (0.89–5.75)	
	0–1 year	8.05 (7.47–8.68)		8.05 (2.64–24.55)	
	1–2 years	7.76 (7.2–8.37)		7.76 (2.47–24.4)	
CP implementation year	2006	1	0.0018	1	0.0312
	2007	1.06 (0.71–1.59)		1.11 (0.77–1.59)	
	2008	0.83 (0.57–1.22)		0.89 (0.63–1.26)	
	2009	1.84 (0.87–3.91)		1.89 (0.9–3.99)	
	2010	1.04 (0.7–1.55)		1.17 (0.9–1.51)	
	2011	1.13 (0.79–1.62)		1.2 (0.83–1.74)	
	2012	0.98 (0.65–1.48)		1.08 (0.74–1.59)	
	2013	2.11 (1.48–3.01)		2.24 (1.62–3.09)	
	2014	1.68 (1.14–2.46)		1.68 (1.18–2.4)	
	2015	1.5 (0.62–3.62)		1.46 (0.56–3.85)	
Age	Min-19	1	0.0018	1	0.0031
	20–29	1.24 (1.09–1.41)		1.27 (1.09–1.47)	
	30–39	1.28 (1.13–1.45)		1.27 (1.09–1.49)	
	40–49	1.26 (1.11–1.42)		1.23 (1.05–1.44)	
	50–59	1.3 (1.15–1.47)		1.26 (1.07–1.47)	
	60–69	1.22 (1.08–1.39)		1.21 (1.02–1.44)	
	≥ 70	1.24 (1.09–1.41)		1.18 (0.95–1.47)	
Gender	Male	1	0.0932	1	
	Female	1.03 (0.99–1.07)		1.04 (1.01–1.06)	
Recommended length of stay		0.99 (0.92–1.06)	0.7996	–	–
Recommended duration of prophylactic antibiotics	1	1	< 0.001	1	< 0.001
	2	0.54 (0.39–0.76)		0.41 (0.26–0.65)	
	3	0.47 (0.35–0.64)		0.44 (0.32–0.62)	

*CI* confidence interval.

^1^p-value calculated using the log likelihood ratio test.

The recommended duration of prophylactic antibiotics was inversely related to the adherence rate, i.e., CP with a longer duration of prophylaxis had a lower adherence rate. However, the implementation year, age, gender, and recommended length of hospitalization did not create a significant impact on the adherence rate (Table 2).

### Effect of CP application and completion on the adherence rate

CP application was associated with a higher adherence rate. The CP applied and completed patients demonstrated a higher adherence rate than their not-applied counterparts in multivariable analysis (IRR 1.94; 95% CI 1.4–2.69) (Table 3). Furthermore, the CP applied but not completed patients showed a higher adherence rate than their not-applied counterparts (IRR 1.54; 95% CI 1.17–2.04).

**Table 3:** Effects of clinical pathway (CP) application and/or completion effect on the adherence rate (n_p_ = 23,469), and the incidence rate ratio (IRR) from a univariable and multivariable mixed-effect Poisson model with random effects.

Variable		Univariable		Multivariable	
		IRR (95% CI)	p-value^1^	IRR (95% CI)	p-value^1^
CP application and completion	Not applied	1	< 0.001	1	< 0.001
	Applied and not completed	1.54 (1.42–1.67)		1.54 (1.17–2.04)	
	Applied and completed	1.94 (1.82–2.07)		1.94 (1.4–2.69)	
Age	> 19	1	0.0753	1	0.2459
	20–29	1.23 (1.07–1.42)		1.19 (1.01–1.4)	
	30–39	1.22 (1.07–1.4)		1.18 (1–1.4)	
	40–49	1.21 (1.06–1.39)		1.16 (0.98–1.37)	
	50–59	1.21 (1.06–1.39)		1.17 (0.99–1.39)	
	60–69	1.18 (1.03–1.36)		1.15 (0.95–1.38)	
	≥ 70	1.15 (1–1.33)		1.13 (0.9–1.4)	
Gender	Male	1	0.1986	1	0.2697
	Female	1.03 (0.99–1.07)		1.02 (1–1.05)	
Recommended duration of prophylactic antibiotics	1	1	0.0106	1	0.0052
	2	0.53 (0.33–0.83)		0.5 (0.3–0.84)	
	3	0.57 (0.38–0.85)		0.58 (0.45–0.74)	

*CI* confidence interval.

^1^p-value calculated using the log likelihood ratio test.

## Discussion

CP implementation increased the adherence to the antibiotic prophylaxis guideline, similar to previous studies. For example, it reduced the prolonged use of prophylactic antibiotics in patients undergoing laparoscopy-assisted vaginal hysterectomy^17^. Furthermore, it increased the rate of administration of prophylactic antibiotics, within 60 min, prior to the incision from 87.4 to 99.2%. Moreover, the rate of discontinuation of prophylactic antibiotics within 48 h increased from 51.6 to 84.7%^18^. CP implementation was associated with less antibiotic use in a retrospective study on patients undergoing endoscopic retrograde cholangiopancreatography for common bile duct stones^19^. However, previous studies examined the effect of CP implementation on the use of prophylactic antibiotic under specific disease conditions. The strength of our study was that it evaluated the effect of CP implementation on adherence to the antibiotic prophylaxis guideline in a cohort including 61 and 44,062 CPs and patients.

CPs were defined diversely in previous studies. Most CPs comprise an algorithm consisting of evaluations, interventions, and standardized care plans. Furthermore, they facilitate an ambiguously defined element, such as ‘routine nursing’^19^. However, the orders in CP should be specific as they are entered into an electronic order entry system. In other words, every CP order is not narrative but a specifically defined element. For example, a CP containing an order of ‘vital sign check every 8 h’ is different from that of ‘vital sign check every 4 h.’

Considering the detailed definition of a CP, the system provided flexibility to a physician to decide about its application on a patient. Therefore, it is recommended not to apply a CP to an unusual patient such as one for whom an intensive care unit admission is anticipated. This can be attributed to the calibration of the usual orders of a CP for an ordinary patient. Therefore, a CP was not applied to some patients, following its implementation into the system. In addition, despite the application, we permitted termination of CPs. For example, CP orders should not be applied to a patient with complications as they were designed for one without complications.

CP application and completion also increased adherence to the prophylaxis guideline. Our study findings are unique in that most previous studies did not distinguish CP implementation from its application. Furthermore, they did not define CP completion^17–20^. Moreover, the strength of the study includes the level of specification (narrative recommendation vs. element-wise systematic order) and the use of the electronic order entry system.

The electronic order entry system allowed a physician to use his or her discretion for not applying CPs to eligible patients. Therefore, our attempts to estimate the CP effect only in patients in whom it was applied could be misleading. A physician might specifically apply a CP to an ‘simple’ patient and use the prophylactic antibiotic for a short duration. However, the CP might not be applied to a ‘complex’ patient^21^. Furthermore, the prophylactic antibiotic might exceed the limit. Therefore, we examined the effects of CP on antibiotic prophylaxis in a cohort including patients on whom it was not applied.

Our study had several limitations. First, implementing CPs alone has limitations for effective antibiotic use control because adherence to guidelines is affected by comprehensive factors such as reminders, education^22^, patient conditions^23^, and organizational constraints^24,25^. Second, the change in the guideline by the National Hospital Evaluation Program or government-driven policies might have affected the adherence rate^26^. These external effects were not incorporated into the model. Third, the institutional guideline permits the use of prophylactic antibiotics over 24 h postoperatively, which is less strict than the external guidelines. Despite estimating the trend of the adherence rate, the overall rate at the institute was presumably higher than that in other institutes. Fourth, we did not evaluate direct clinical outcomes, such as changes in the SSI rates. Finally, because this study was conducted in a single center, the results may have been influenced by the specific hospital setting.

Antibiotic stewardship programs aim to optimize antibiotic use and ultimately enhance patient care by combining activities such as CP implementation, intervention, tracking, reporting, and education. In this study, we compared the antibiotic adherence rates before and after CP implementation in an EHR system. The adjusted adherence rate was 8% before CP was implemented, after which it increased to > 60% within two years. When measured during the same time frame, the adjusted adherence rate after CP use was 1.5 times as high as that before CP. The results of this study suggest that a computer-assisted CP system for EHR could improve antibiotic adherence without significant expense.

## Methods

### Clinical pathway development and implementation in an electronic order entry system

This study was conducted at a tertiary university hospital in the Seoul metropolitan area of Korea. The hospital developed a high-level EHR system. Furthermore, it operates a comprehensive system comprising more than 264 CPs as of 2020^27^. This single-site time series was set at the office of the Quality improvement and Process, an institutional government in charge of CP development and monitoring.

CP typically comprises detailed sets of orders that describe how a patient should be cared for, from the time of admission to discharge. CP includes orders on vital signs, activity, diet, intervention, personalized frequency and dosage of medication, fluid administration based on height and weight, and laboratory tests. However, it does not generally contain intraoperative and anesthesia orders.

The dose and duration of prophylactic antibiotics for each CP are tailored down to a specific surgery type. For example, an intravenous injection of 2 g cefazolin is recommended in most CPs; however, 1 g cefotetan is used in the CP for a large bowel resection.

This predetermined dose and duration of prophylactic antibiotics are guided by the Antibiotic Stewardship Program (ASP) of the Seoul National University Bundang Hospital. This consists of a multidisciplinary group, including infectious disease specialists and physicians from various departments. The ASP aims to optimize the use of antibiotics and prevents the overuse of prophylactic antibiotics. Prophylactic antibiotics were previously administered more than a week. The ASP recommended gradually decreasing the duration from 5 to 3 and 2 days. It currently allows the use of prophylactic antibiotics for more than 24 h only if the physicians insist on a prolonged use under certain clinical situations.

A physician or the entire medical department may propose a new CP of detailed orders for a list of eligible surgeries. The proposed CP is reviewed by a multidisciplinary team, including infection specialists, nursing team members, insurance/reimbursement team members, and other physicians, before being implemented into the electronic order entry system.

Most CPs are developed in a hierarchical fashion such that a CP for an upper level surgery can be used interchangeably for a subcategory of the surgery. For example, a list for a CP named ‘laparoscopic hysterectomy’ could include surgery names, such as ‘total laparoscopic hysterectomy’ or ‘laparoscopy-assisted vaginal hysterectomy.’ A physician can apply the ‘laparoscopic hysterectomy’ CP to the patient having ‘total laparoscopic hysterectomy’ or ‘laparoscopy-assisted vaginal hysterectomy’ as the scheduled surgery name.

Before 2016, a physician had to manually assign a CP to a patient. For example, to assign a CP to a patient expected to undergo 'total laparoscopic hysterectomy,’ the physician had to open a screen containing the list of all CPs and select ‘laparoscopic hysterectomy.’ The system was flexible enough that physicians did not have to prescribe a CP, for example, a patient was reserved to be admitted to the intensive care unit postoperatively.

A new system was launched in 2016, which recommends CPs based on the surgery scheduled for each patient. It generates a list of the recommended CPs for each patient. Upon the authorization of an electronic signature by a physician, the chosen CP is activated. This is followed by an entry of the orders of the selected CP into the EHR system. Therefore, the study period was set from 2006 to 2015 to exclude the system-specific effect.

Physicians can modify the preselected orders of CP adapted to a patient situation, if necessary. For example, physicians prescribe other antibiotic medications based on their experience of a patient with fever of an unknown cause with suspected infection. In addition, they can stop applying a CP for various reasons, such as development of postoperative ileus or inability to consume the scheduled diet. Furthermore, CP application is automatically terminated when the duration of admission surpasses the predefined limit. Therefore, the completion of a CP refers to the status that the CP is terminated on schedule; however, it allows for a maximum difference of 2 days.

The monitoring team organized at the office of the Quality improvement and Process defined and monitored the institution-wide metrics for CP usage. The application rate is defined as the number of CP applied patients divided by the number of CP eligible patients (a person who underwent a surgery from the list of eligible surgeries). The completion rate is defined as the proportion of patients who completed a CP among those for whom CP was applied. The monitoring team investigates the causes and provides feedback to the associated department on a quarterly basis on observing a low application or completion rate.

### Study population

Our study protocol was approved by the Seoul National University Bundang Hospital institutional review board (B-1612-375-105) and the study methods were carried out in accordance with the Declaration of Helsinki. Furthermore, the need for informed consent was waived off by the aforementioned institutional review board. We selected all 175 CPs with available metrics for an elective surgery. We excluded CPs of an insufficient number of patients (at least 20 patients, 2 years before and after each CP implementation), short period of observation (< 2 years before and after each CP implementation) (n_c_ = 80), CPs without an antibiotic prophylaxis (n_c_ = 13), and those with an extremely high or low adherence rate (< 10% or > 90%) (n_c_ = 21). Therefore, a total of 61 CPs were eligible for this study.

We selected patients who met the following criteria: (1) included in one of the 61 CPs and (2) admitted between 2 years, before and after the implementation date of each CP (Fig. 1).

### Objectives and variables

**Figure 3:**
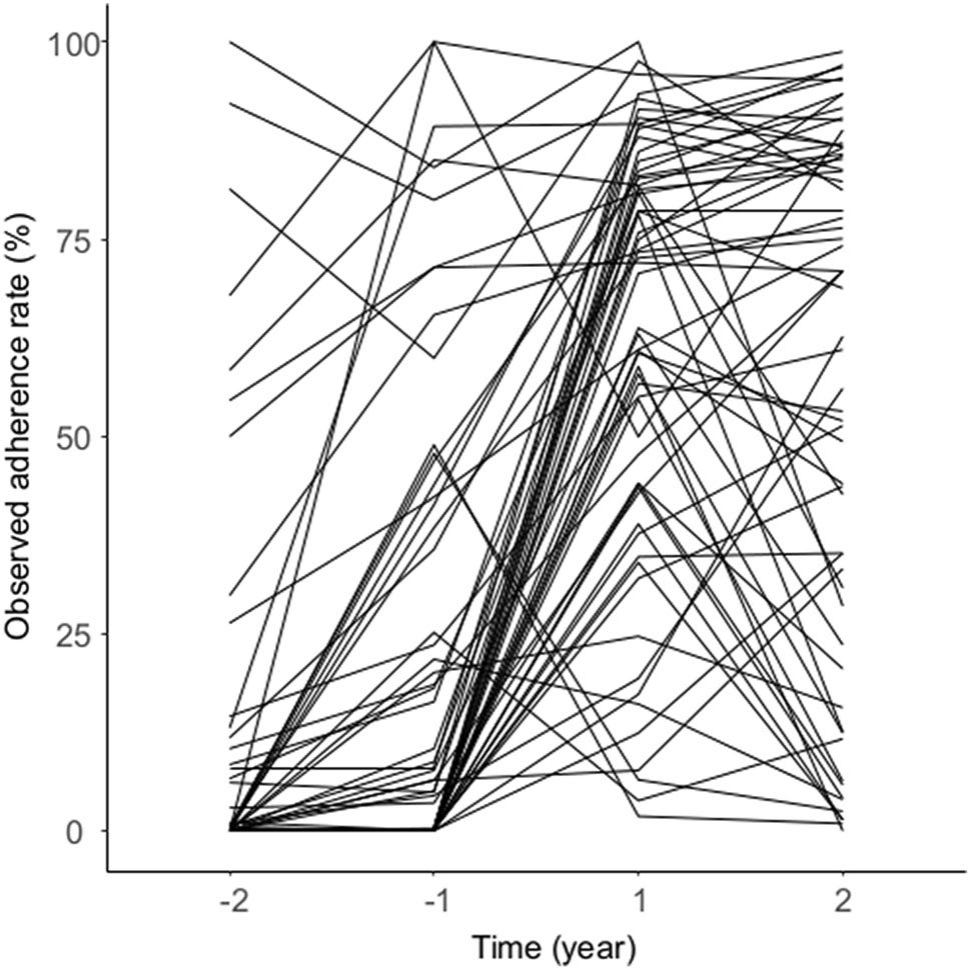
Data analysis frame for the primary (clinical pathway [CP] implementation effect) and secondary (CP application and/or completion effect) objectives.

Our primary objective was to examine the effect of CP implementation on adherence to the antibiotic prophylaxis guideline recommended by ASP. Our secondary objectives were to evaluate the effect of CP application and completion on adherence to the aforementioned guideline (Fig. 3).

The number of eligible patients were abstracted for each CP along with implementation year, recommended length of stay, and recommended duration of antibiotic prophylaxis.

We collected information on age, gender, admission date relative to the CP implementation date, duration of prophylactic antibiotics, eligible CP name, and whether the CP was applied and/or completed for the patient-level data. The admission date relative to the CP implementation date was defined as the difference between the admission date and the CP implementation date. For example, a patient was admitted on January 1, 2010 for 'total laparoscopic hysterectomy'. The CP was not developed for 'total laparoscopic hysterectomy' in 2010. Therefore, the patient received usual care. The CP of ‘laparoscopic hysterectomy’ was developed and implemented in the electronic order entry system on January 1, 2011. The eligible list of surgeries for the CP of ‘laparoscopic hysterectomy’ contained ‘total laparoscopic hysterectomy.’ Therefore, the admission date relative to the CP implementation date was − 1 year for the patient. In contrast, the relative date was + 4 months for another patient admitted on January 5, 2011 for ‘total laparoscopic hysterectomy.’

We used both dosage and the duration of prophylactic antibiotics to determine adherence to the above-mentioned guideline for each patient. Adherence was defined as the use of a CP-recommended antibiotic within the permitted duration. In contrast, the use of other antibiotics or the prolonged use of antibiotics indicated non-adherence. However, we did not include the timing of the antibiotic administration (within 60 min, prior to the surgical incision) in the analysis.

### Statistical analysis

We used a mixed-effect piecewise Poisson regression with a random slope to model the adherence rate^28^. Despite the binary adherence outcome, we selected a Poisson model with robust variance estimators because of the high rate of overall adherence. We established a core part of the mixed model for the *i*th CP as follows to assess the effect of CP implementation:

$$\mathrm{log}\left({\mu }_{ti}\right)= {\beta }_{i0}+{\beta }_{1}{T}_{ti}+{\beta }_{2}{I}_{i}+{\beta }_{3}I{T}_{ti}.$$

In the aforementioned equation, ${\beta }_{i0}$ estimates intercept, ${\beta }_{1}$ estimates the baseline trend pre-implementation, ${\beta }_{2}$ estimates the overall change in adherence post-implementation, and ${\beta }_{3}$ estimates the incremental change in the trend post-implementation. The relative admission date to the CP implementation was denoted as ${T}_{ti}$. It was recoded as a four-level categorical variable with the following levels: − 2 year ~ − 1 year, − 1 year ~ CP implementation, CP implementation ~  + 1 year, and + 1 ~  + 2 year. The confounders included age, recommended length of hospitalization, and recommended duration of prophylaxis. The aforementioned model produced the incidence rate ratios (IRRs). We examined that the effect of CP implementation on the adherence rate lasted for a long term. This was followed by testing the CP application and/or completion effect by a cross-sectional data analysis. It eventually omitted the time variable from the model:

$$\mathrm{log}\left({\mu }_{i}\right)= {\alpha }_{i0}+{\alpha }_{1}{C}_{i}.$$

The model included the effect of CP attempted and not completed and the effect of CP completed ($$C)$$. We performed data cleaning using Python 3.6.9. The statistical modeling was conducted STATA version 15 (College Station, TX, USA) and R 3.6.3 at a two-sided alpha level of 0.05.

## Data Availability

The data that support the findings of this study are available from the corresponding author upon reasonable request.
